# Retracted: Chemical Speciation and Potential Mobility of Heavy Metals in the Soil of Former Tin Mining Catchment

**DOI:** 10.1155/2018/5780515

**Published:** 2018-12-12

**Authors:** The Scientific World Journal

At the request of the authors, the article titled “Chemical Speciation and Potential Mobility of Heavy Metals in the Soil of Former Tin Mining Catchment” [1] has been retracted. The article has a high similarity index.
